# The impact of pesticides used at the agricultural land of the Puck commune on the environment of the Puck Bay

**DOI:** 10.7717/peerj.8789

**Published:** 2020-03-19

**Authors:** Grażyna Pazikowska-Sapota, Katarzyna Galer-Tatarowicz, Grażyna Dembska, Marta Wojtkiewicz, Ewelina Duljas, Stefan Pietrzak, Lidia Anita Dzierzbicka-Glowacka

**Affiliations:** 1 Department of Environment Protection, Gdynia Maritime University, Maritime Institute, Gdynia, Poland; 2 Department of Water Quality, Institute of Technology and Life Sciences in Falenty, Falenty, Poland; 3 Physical Oceanography Department, Institute of Oceanology of the Polish Academy of Science, Sopot, Poland

**Keywords:** Pesticides, Active substances, Surface runoff, Groundwater, Agricultural land, Sediments, Fish, Puck Bay

## Abstract

**Background:**

The Puck commune is one of the largest agricultural regions in the Pomeranian Voivodship that due to the pollution of the coastal zone negatively affects the functioning of the Puck Bay, including health of its inhabitants, and causes decrease in tourism as well as in overall economic value of the region. The objective of the undertaken study was to assess the extent of risk to the environment posed by the pesticides used in agricultural production in the coastal area of the Puck commune.

**Methods:**

The study focused on organochlorine insecticides (DDT and its metabolites: α, β, ϒ, δ-HCH, aldrin, dieldrin, endrin, isodrine), glyphosate and its metabolite AMPA, and 309 active substances used as pesticides. Analyses were carried out using GC-MS, GC-MS/MS and LC-MS/MS techniques. The undertaken novel approach included “tracking” of a large number of substances in multiple environmental matrices (surface water, groundwater, seawater, soil, sediment and fish), along with examination of their transport routes from the pesticides application locality to the Puck Bay.

**Results:**

Glyphosate and its metabolite AMPA, anthraquinone, boscalid, chlorpyrifos-ethyl, dimethachlor, diflufenican, difenoconazole, epoxiconazole, fluopicolide and metazachlor were found in soil samples and surface water samples collected from drainage ditches surrounding the studied agricultural plots. In the samples of seawater and fish taken from the Puck Bay no studied pesticides were found.

## Introduction

The Baltic Sea is a brackish, shallow, semi-enclosed shelf sea, with an inflow of the North Sea waters limited by three straits, i.e., Oresund, the Great Belt and the Little Belt. Its water density stratification and thermal stratification are both pronounced, which hinders mixing of well oxygenated surface waters with poorly oxygenated deep waters. Numerous rivers discharge into the Baltic Sea, which is the main reason for positive freshwater balance, where freshwater input exceeds evaporation (Lomniewski, Mańkowski & Zaleski, 1975; Cyberska, 1990; Gerlach, 1994; Andrulewicz et al., 2008). The aforementioned specific characteristics expose the Baltic Sea to strong anthropopressure resulting from extensive demographic and economic development of nine riparian countries (Andrulewicz et al., 2008).

The Puck Bay is a unique part of the Baltic Sea, also called the “Little Sea”. The Bay is 364 km^2^, with the maximum depth of 55 m (Chałupska Jama), but a significant part of it is very shallow. The Puck Bay borders the Hel Peninsula in the north, the Gulf of Gdansk in the east, and the North Kashubian coastline in the south-west. The Puck Bay is an attractive region and a perfect place for tourism, recreation, water sports and fishing. However, the Puck commune area is one of the largest agricultural regions in the Pomeranian Voivodship that due to the pollution in the coastal zone negatively affects functioning of the entire environment, including human health, and results in decrease in tourism and in overall economic value of the region. EU rules distinguish between active substances, such as glyphosate, and plant protection products. Plant protection products (PPP)—which are often referred to as pesticides (e.g., insecticides, fungicides, herbicides)—are mixtures containing one or several active substance(s) and other ingredients (so-called co-formulants) (European Commission, 2017a; European Commission, 2017b; Pérez-Lucas et al., 2018). Pesticides widely used in agriculture, horticulture and forestry, pose a significant threat to the environment (Karami-Mohajeri & Abdollahi, 2010; Rodney, Teed & Moore, 2013; Lari et al., 2014; Sidoli, Baran & Angulo-Jaramillo, 2016; Bento et al., 2017; Erban et al., 2018; Lupi et al., 2019; Shang et al., 2019). The use of pesticides in agriculture has help to improve yields by preventing crop losses. Pesticides include active ingredients that in spite of the beneficial actions on agricultural production could have other less positive impacts on the environment and habitats where they are used (Eurostat, 2016; Shang et al., 2019), e.g., they can contaminate food, air, soil and water (Grygiel et al., 2012; Żak, 2016; Bento et al., 2017). Pesticide toxicity results from the presence of biologically active ingredients, emulsifiers, excipients and fillers that may adversely affect environmental biocenosis (Nowak, Włodarczyk-Makuła & Mamzer, 2015). About 10,000 products of this type that differ in their purpose, composition and properties are known (Sadecka, 2003). Pesticides may undergo physical and/or chemical changes into the environment and also be transferred between different ecosystems as original compound or as product of degradation/metabolism. In the initial form or as a derivative, their metabolites have the ability to retention in soil, water, atmosphere, as well as human food and animal feed threatening wellbeing of all living organisms (Żelechowska, Biziuk & Wiergowski, 2001). Based on their function pesticides are divided into: fungicides, insecticides (protect plants against the harmful effects of insects) and herbicides. Pesticides are also classified based on their persistence in the environment and categorized as: unstable (spread out up to 12 weeks), moderately persistent (spread out in 1–18 months) and highly persistent (spread out within 2–3 years in 75–100%) (Nowak, Włodarczyk-Makuła & Mamzer, 2015). The last group includes organochlorine pesticides (DDT, HCH, aldrin and others), the use of which has already been banned, however, many years of research show that their residues still persist in the Baltic Sea both in inanimate matter (bottom sediments) and in the tissues of living plants and animals at all trophic levels (Backlund, Holmbom & Lepakoski, 1992; Singh & Agarwal, 1995; Wodarg & Graeve, 1996; Sapota, 2006a; Sapota, 2006b; Sapota & Wiśniewska-Wojtasik, 2007; Sapota et al., 2009; Staniszewska et al., 2011). It has been proven that the use of pesticides diminishes the agriculture losses, which is associated with economic growth. On the other hand, they may pose a serious threat to human health. It is true not only for individual active substances, but also for the components of preparations in which various chemical compounds “facilitate” practical use (Makles & Domański, 2008; STOA, 2019). Therefore, the new approach to agriculture and horticulture focuses on the introduction of new, resistant plant varieties, which significantly reduce the risk of diseases and pests and make the use of pesticides largely unnecessary (Calvert, 2019; Cary, 2019). However, the negative pressure of agriculture on the environment is still very strong. It means that recently the market of plant protection chemicals has been changing significantly and so it will in the near future, because further active substances are being withdrawn and pesticides with lower toxicity are introduced instead. The obligation to exercise control over the use of pesticides and plant protection products in agricultural production, including testing for residues of active substances of plant protection products, results both from national law, in particular the act on plant protection products (Journal of Laws of 2013, item 455 with later amendments), as well as from the European Union regulations (Miszczak, 2016; STOA, 2019). Directive 2009/128/EC establishing a framework for Community action for the sustainable use of pesticides (Official Journal of the European Union L 309, 24.11.2009, page 71) regulates at the level of the European Union the rules of trading and use of plant protection products, in order to reduce the risks to human health, animals and the natural environment posed by these preparations. In EU countries, the conditions for the registration approval of a given pesticide and its placing on the market are determination of the effectiveness of the preparation and assessment of the risks posed to humans and the environment. The only substances allowed for distribution are the ones for which tests have shown little effect on human health and the environment. If high risk is detected, actions are taken to reduce or to prohibit the use of the pesticide (Nowak, Włodarczyk-Makuła & Mamzer, 2015; Pérez-Lucas et al., 2018). In an action aiming to reduce pesticide environmental hazards, the European Union recommends a strategy for sustainable use of pesticides, through increased use and distribution controls, introduction of intensive training of farmers in maintaining proper dosing of pesticides, keeping records of spraying, types of substances used and type of crops, using certified spraying devices, or “pesticide” taxation of agrochemical companies, used to cover the costs of permit issuance, of inspections and testing as well as training (Makles & Domański, 2008; European Commission, 2017a). The objective of this study was to assess the extent of risk pesticides used in agricultural production in the coastal area of the Puck commune pose to the environment. For this purpose, the content of pesticides in arable lands as well as in water samples collected in selected watercourses and drainage ditches surrounding the investigated agricultural parcels in the area of the Puck commune, and also in samples of seawater, sediments and fish muscle were examined. When selecting agricultural land plots for research, the following criteria were taken into account: location of arable land in relation to watercourses, type of crops, application of specific pesticides and plant protection products. The information was derived from a questionnaire presented to farmers participating in the project titled “Modelling of the impact of the agricultural holdings and land-use structure on the quality of inland and coastal waters of the Baltic Sea set up on the example of the Municipality of Puck region—Integrated info-prediction Web Service WaterPUCK” (Dzierzbicka-Głowacka et al., 2019). The study covered organochlorine insecticides (DDT and its metabolites: *α*, *β*, υ, *δ*-HCH, aldrin, dieldrin, endrin, isodrin). At the same time, based on the information provided in the characteristic’s sheets of the declared plant protection products, a list of active substances with increased likelihood of leaving their residues in the environment and substances with a long half-life in the environment was established. 309 compounds classified as pesticides were included in this list. The novel approach undertaken in the study was “tracking” of a large number of substances in a number of environmental matrices, along with examination of their transport routes from their application place to the Puck Bay.

## Materials & Methods

### Sampling

**Figure 1:**
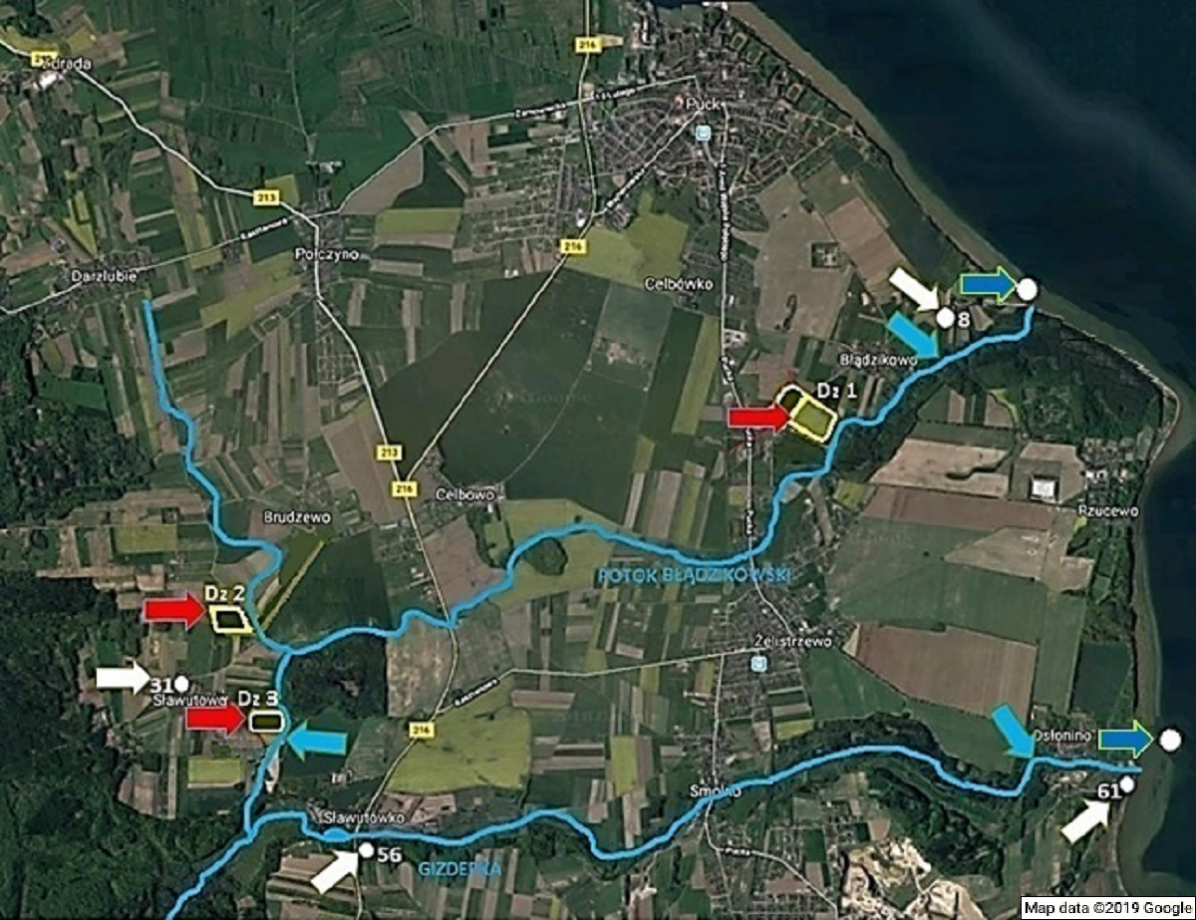
Location of sampling sites. Map data ©2019 Google. (A) The red arrow indicates sampling sites of soil and surface water from drainage ditches. (B) The white arrow indicates sampling stations of groundwater. (C) The light blue arrow indicates sampling stations of surface water from watercourses. (D) The blue arrow indicates sampling stations of surface seawater and sediment.

Analyses of the content of pesticides in watercourses and arable lands were carried out in the area of the agricultural Puck commune, located in the north-eastern region of the Pomeranian Voivodeship, on the west coast of the Puck Bay, which is part of the Baltic Sea. Agricultural land in the Puck commune constitutes 57.3% of the total area of the region, the vast majority of which is characterized by high crops potential. The area landscape is largely undulating, with land slopes of up to approx. 9% (5.14°). Such terrain characteristics are conducive to leaching of the applied plant protection products from soils as a result of surface runoff. Sampling was carried out with the consent of the Puck commune and farmers who took part in the project. Figure 1 shows the points and areas for sampling.

The study covered three agricultural land plots belonging to two farms participating in the WaterPUCK project which were marked as G1 and G2. One of the plots number Dz 1, belonged to the G1 farm, and two plots, number Dz 2 and Dz 3, to the G2 farm. Selection of farms was made taking into account location of arable land relative to watercourses and the size of the average consumption of active substances of plant protection products declared in the questionnaire in relation to the arable land area. On the studied plots occurred mainly brown soils (Pietrzak et al., 2020). In terms of the agronomic category the soils belonged to medium soils, slightly acidic.

Soil samples for chemical analyses were collected in 2018 during the intensive application of pesticides (in March, April and June) and after the use of it (in July and August). According to standard PN-R-04031:1977, 60 general samples (averaged) from the soil surface layer (0–30 cm) were collected. For one general sample, up to 20 single samples were taken uniformly from the surface of the entire field.

Water samples were collected once a month from February to September 2018 from:

- drainage ditches surrounding each parcel (56 surface water samples)

- watercourses receiving water from the drainage ditches investigated and then flowing to the Puck Bay, i.e., the Bladzikowski Stream; the channel connecting the Bladzikowski Stream with the Gizdebka River; the Gizdebka River (21 surface water samples)

- seawater from the Puck Bay (two points in the mouths of the studied watercourses) (16 sample of surface water)

and:

- four piezometers: No. 8, 31, 56 and 61 (4 samples of groundwater taken in August 2018).

Bottom sediment samples were taken with van Veen grab in the same points as seawater samples (16 samples of surface layer).

**Figure 2:**
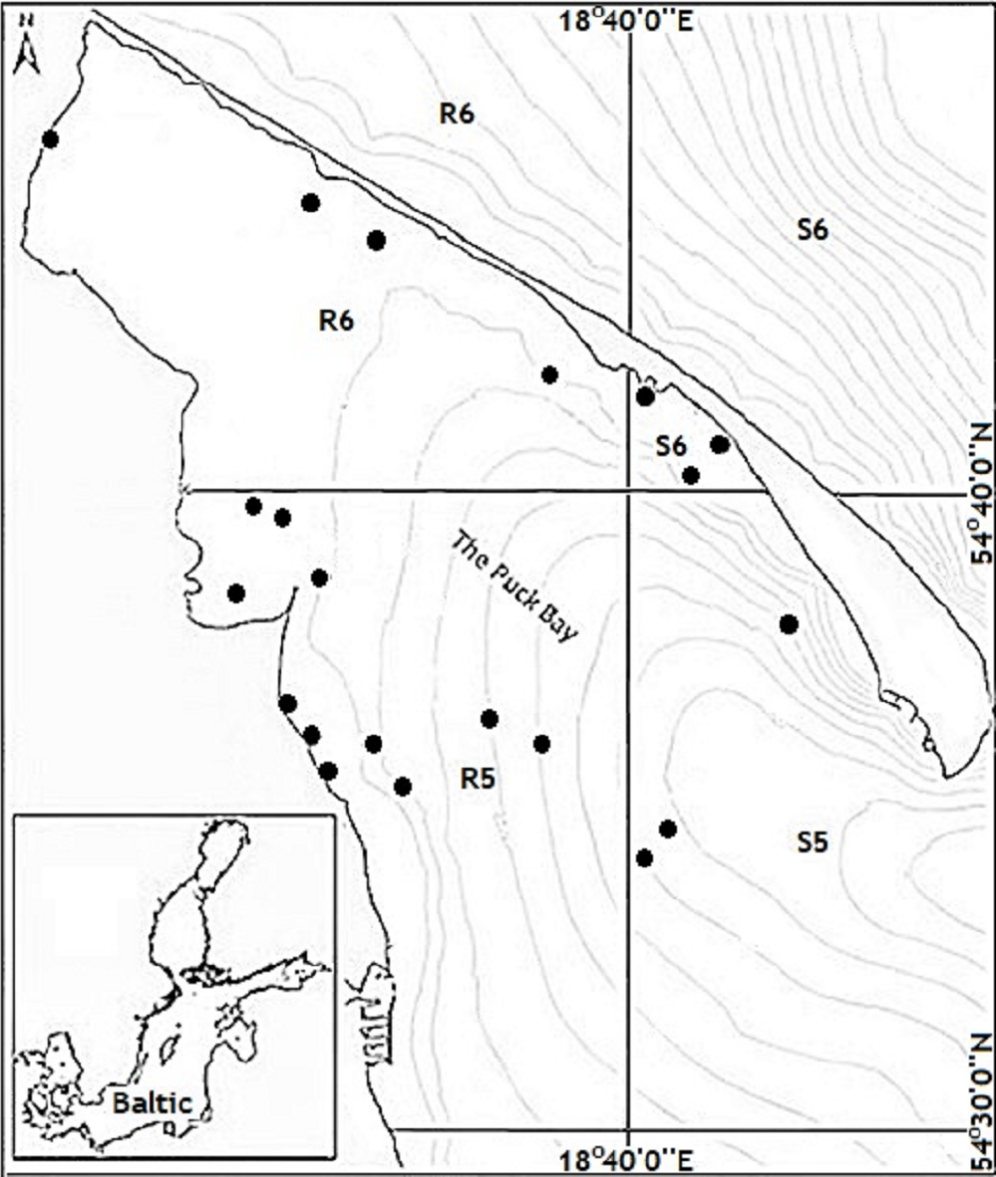
Sites of fish catching.

Research fishing was carried out using the “MEC-8” fishing unit and the Maritime Institute boat “BOSMINA II”. The spatial range included fishing squares R-5, R-6, S-5, S-6 (Fig. 2) within the boundaries of the Puck Bay. A total of 60 catch using the NORDIC multi-panel gillnet set were carried out from May to the end of October. Two species of fish, cod (*Gadus morhua*) and flounder (*Platichthys flesus*), were selected for the study. 25 specimens of each species were taken for testing of the pesticides content.

### Analyses

All samples: soils, sediments, surface and ground waters and fish muscle have been tested for the presence of organochlorine insecticides (aldrin, dieldrin, endrin, isodrine, sum of DDT, hexachlorocyclohexane (HCH): alpha, beta, gamma and delta); herbicide glyphosate and its metabolite AMPA (aminomethylphosphonic acid); and 309 active substances of plant protection products (Table 1). Both active substances, which according to the regulations are tested in environmental samples, as well as those currently examined only as residues in food were analysed. The lower limits of quantification of individual compounds were below the environmental quality standards (EQS) set out in the Regulation of the Minister of the Environment of July 21, 2016 *on the method of classification of the surface water bodies and environmental quality standards for priority substances* (Journal of Laws 2016 poz.1187), as well as below the Maximum Residues Level of an active substance in food (MRL) defined in the EC Regulation No. 396/2005 of the European Parliament and of the Council of 23 February 2005 *on maximum residue levels of pesticides in food and feed of plant and animal origin and on their surface.*

**Table 1:** List of studied active substances of plant protection products.

**No.**	**The active substance**	**No.**	**The active substance**	**No.**	**The active substance**	**No.**	**The active substance**
1	2,4,5-T-Methylester	78	4,4-Dibromobenzophenone	155	Fluopicolide	232	Pentachloraniline
2	2,4-D-Methylester	79	Dicapthon	156	Fluorodifen	233	Pentachlorobenzene
3	Acetochlor	80	Dichlobenil	157	Fluotrimazole	234	Pentachlorothioanisole
4	Aclonifen	81	Dichlofenthion	158	Fluchinconazole	235	Permethrin
5	Acrinathrin	82	Dichlofluanid	159	Flurenol-buthyl	236	Pertan
6	Alachlor	83	Dichloran	160	Flurochloridone	237	Phenkapton
7	Aldrin+Dieldrin	84	Dichlorvos	161	Flurtamone	238	Phenothrin
8	Allethrin	85	Diclofop-methyl	162	Flusilazole	239	Phenthoate
9	Amidithion	86	Dicofol (sum)	163	Folpet	240	Phosalone
10	Anthraquinone	87	Dicofol,o,p-	164	Fonofos	241	Phosfolane
11	Atrazine	88	Dicofol,p,p–	165	Formotion	242	Phosmet
12	Azaconazole	89	Dicrotophos	166	Genite	243	Picolinafen
13	Azinophos ethyl	90	Dienochlor	167	Halfenprox	244	Picoxystrobin
14	Azinophos methyl	91	Difenoconazole	168	Haloxyfop-Ethoxyethyl	245	Piperophos
15	Azoxystrobin	92	Diflufenican	169	Haloxyfop-methyl	246	Pirymifos methyl
16	Benfluralin	93	Dimefox	170	HCH (sum of isomers - without lindane)	247	Pirymifos ethyl
17	Benoxacor	94	Dimetrachlor	171	Epsilon-HCH	248	Plifenate
18	Benzoylprop-ethyl	95	Dimethipine	172	Lindane	249	Prallethrin
19	Biofenox	96	Dimethoate	173	Heptachlor	250	Procymidone
20	Binapacryl	97	Dimethomorph	174	Heptachlor (sum)	251	Profenfos
21	Bifenthrin	98	Diniconazole	175	Heptachlor-trans-epoxide	252	Profluralin
22	Bitertanol	99	Dinitramine	176	Heptachlor-cis-epoxide	253	Propachlor
23	Boscalid	100	Dinobuton	177	Heptenophos	254	Propanil
24	Bromofenvinphos	101	Disulfoton	178	Heksaconazole	255	Propazine
25	Bromocyclen	102	Disulfoton-sulfone	179	Indanofan	256	Propetamphos
26	Bromophos methyl	103	Ditalimfos	180	Indoxacarb	257	Propiconazole
27	Bromophos ethyl	104	Edifenphos	181	Jodofenfos	258	Propyzamide
28	Bromopropylate	105	Endosulfan (sum of isomers)	182	Ioxynil-Octanoate	259	Prothiofos
29	Buprofezin	106	alpha-Endosulfan	183	Iprobenfos	260	Prothoat
30	Butachlor	107	Endosulfan sulphate	184	Iprodion	261	Pyraclofos
31	Butamifos	108	beta-Endosulfan	185	Isazophos	262	Pyraflufen-ethyl
32	Butralin	109	Ketoendrin-delta	186	Isobenzan	263	Pyrazophos
33	Cadusafos	110	EPN	187	Isocarbofos	264	Pyrethrins
34	Captafol	111	Epoxiconazole	188	Izofenfos	265	Pirydaben
35	Captan	112	Etaconazole	189	Isofenphos-Methyl	266	Pyridaphenthion
36	Carbophenothion	113	Ethalfluralin	190	Isomethiozin	267	Pyrifenox
37	Carbophenothion-methyl	114	Ethion	191	Isopropalin	268	Pirimithate
38	Carfentrazone-ethyl	115	Ethiprol	192	Isoxadifen-ethyl	269	Chinalofos
39	Chinomethionat	116	Ethofumesate	193	Kresoxim-metylu	270	Qinoxyfen
40	Chlorbenzyd	117	Ethoprophos	194	Lactofen	271	Quintozene
41	Chlordane (total)	118	Etridiazole	195	Leptophos	272	Quintozene (sum)
42	Chlordan,cis-	119	Etrimfos	196	Lufenuron	273	Chisalofop ethyl
43	Chlordan,oxy-	120	Famophos	197	Malaoxon	274	Resmethrin
44	Chlordan, gamma/trans-	121	Famozadone	198	Malathion	275	S421 Octachlordipropyleter
45	Chlordecon	122	Fenamidone	199	Mekarbam	276	Spiromesifen
46	Chlorethoksyfos	123	Fenamiphos (sum of isomers)	200	Mephosfolan	277	Sulfotep
47	Chlorfenapyr	124	Fenarimol	201	Merphos	278	Sulprofos
48	Chlorfenprop-methyl	125	Fenbuconazole	202	Metazachlor	279	Swep
49	Chlorfenson	126	Fenchlorazole	203	Metakrifos	280	tau-Fluvalinate
50	Chlorfenvinphos	127	Fenchlorofos	204	Methidation	281	Tebupirimfos
51	Chloridazon	128	Fenfluthrine	205	Metoxychlor	282	Tecnazene
52	Chlormephos	129	Fenhexamid	206	Metolachlor	283	Tefluthrin
53	Chlorobenzilate	130	Fenitrothion	207	Metrafenone	284	Temephos
54	Chloroneb	131	Fenoxaprop-ethyl	208	Metribuzin	285	Terbufos
55	Chloropropylat	132	Fenpiclonil	209	Mevinfos	286	Tetrachlorvinphos
56	Chlorothalonil	133	Fenpropathrin	210	Mirex	287	Tetraconazole
57	Chlorpyrifos ethyl	134	Fenpropimorph	211	Molinate	288	Tetradifon
58	Chlorpyrifos methyl	135	Fenson	212	Myclobutanil	289	Tetramethrin
59	Chlorthal-dimethyl	136	Fensulfothion	213	Nitralin	290	Tetrasul
60	Chlorotion	137	Fevalerate (sum of isomers)	214	Nitrapyrin	291	Tolklofos methyl
61	Chlortiofos	138	Fenvalerate	215	Nitrofen	292	Tolylfluanid
62	Chlozolinate	139	Fenvalerate(RS-/SR-Isomer)	216	Nitrothal-isopropyl	293	Toxaphene Parlar 26
63	Cinidon-ethyl	140	Fipronil	217	Norflurazon	294	Toxaphene Parlar 50
64	Clodinafop-propargyl	141	Fipronil, desulfinyl-	218	Nuarimol	295	Toxaphene Parlar 62
65	Cumafos	142	Fipranil+Sulfonmetab. MB46136 (sum)	219	Ometoate	296	Transfluthrin
66	Crotoxyphos	143	Fipronil sulfide	220	Oxadiazon	297	Triadimefon
67	Cyjanofenphos	144	Fipronil sulfone	221	Oxydemeton-methyl	298	Triadimenol
68	Cyanophos	145	Flamprop-isopropyl	222	Oksyfluoroten	299	Triadimenol/Triadimefon (sum)
69	Cyfluthrin	146	Flamprop-methyl	223	Paclobutrazole	300	Triallate
70	lambda-Cyhalothrin	147	Flonicamid	224	Paraoxon-ethyl	301	Triamiphos
71	Cypermethrin	148	Fluazifop-buthyl	225	Paraoxon-methyl	302	Triazophos
72	Cyphenothrin	149	Fluazinam	226	Parathion	303	Tribuphos
73	Cyproconazole	150	Fluchloralin	227	Parathion methyl	304	Trichloronate
74	Deltamethrin	151	Flucythrynate	228	Parathion methyl/Paraoxon methyl (sum)	305	Tridiphane
75	Dialifos	152	Flufenoxuron	229	Penconazole	306	Trifloxystrobin
76	Di-allate	153	Flumethrine	230	Pendimethalin	307	Trifluralin
77	Diazinon	154	Flumetralin	231	Pentachloranisol	308	Vamidothion
						309	Vinclozolin

### Water samples

The determination of chloroorganic pesticides residue in water samples had been performed following PN-EN 16693: 2015-12 standard *Water quality—Determination of selected chloroorganic pesticides (OCP) in whole water samples—Solid phase extraction method (SPE) using extraction discs and gas chromatography with mass spectrometry (GC-MS).* To 1 dm^3^ of water sample was adding internal standard (e.g., chrysene D12, Accu Standard). The entire sample volume was transferred through speed disc filled by C18 (SPE, J.T. Backer) and then washed by dichloromethane acetone (Merck for GC-MS SupraSolv) 1:1 (v/v). The sample was reconstituted to 1 cm^3^ and measured by GC-MS. LOQ depending on pesticide was 0.005–0.01 µg dm^−3^.

The examination of the content of 309 active substances classified as pesticides, glyphosate and AMPA in water samples had been performed in accordance with the methodology contained in SOP-LA-GC-015-06. The 309 active substances were liquid/liquid extracted from 30 cm^3^ of water sample using mixture of n-hexane/toluene/ethyl acetate (Merck for GC-MS SupraSolv). The organic solvents were evaporated to dryness. Dry residue were resolved in 300 mm^3^ of ethyl acetate and measured using GC-MS/MS. LOQ depending on pesticide ranged from 0.02 to 0.10 µg dm^−3^.

The glyphosate and AMPA in water samples was analysed after sample homogenization and pre-column derivatization on line with 9-fluorenylmethyl chloroformate (FMOC, Sigma-Aldrich) (pH 9), and chromatographic column separation. Finale analyses had been performed on LC-MS/MS. LOQ was 0.02 µg dm^−3^.

### Soil and sediment samples

The determination of chloroorganic pesticides content in soil and sediment samples had been performed according own research procedure PB-45 “Determination of organochlorine pesticides in soil samples”, edition No. 2. Pesticides were extracted from 10 g of dry soil or sediment sample with 50 cm^3^ n-hexane/acetone (Merck for GC-MS SupraSolv) 1:1 (v/v) in ASE (Accelerated Solvent Extraction). After cleaning with sulphuric acid (Merck, Suprapur) on the column filled by silica gel (J.T. Baker) and evaporating to 1 cm^3^, chloroorganic pesticides were measured using GC-MS. LOQ for each pesticide was 0.02 mg kg^−1^.

The examination of the content of 309 active substances classified as pesticides was carried out in accordance with the methodology described in SOP-LA-GC-033-04 and D. Becker 1.4.2019. The active substances were extracted from 5 g of dry soil or sediment sample with water, acetone, n-hexane (Merck for GC-MS SupraSolv) and sodium chloride (Sigma-Aldrich). After shaking (0.5 h) and centrifuging, 1 cm^3^ of supernatant was transferred into vial and measured using GC-MS/MS. LOQ depending on pesticide ranged from 0.015 to 0.12 mg kg^−1^.

The content of glyphosate and AMPA had been performed following SOP-LA-LCMS-039-04 and D. Becker 1.4.2019. The 2 g of dry soil or sediment sample was extracted with 10 cm^3^ KOH (Supelco). After neutralising and centrifuging the samples, 1 cm^3^ of the supernatant was transferred to a plastic tube. Isotopic marked internal standard was adding and then derivatization step was carried out with FMOC-Cl (Sigma-Aldrich) and acidation to improve retention and LC-MS/MS detection. LOQ was 0.05 mg ⋅kg^−1^.

### Fish

The determination of chloroorganic pesticides content in muscle tissue of fish had been performed according own research procedure PB-46 “Determination of organochlorine pesticides in biota samples”, edition No. 2.

Pesticides were extracted from 2 g of dry (lyophilised) tissue with 20 cm^3^ n-hexane/acetone/dichloromethane (Merck for GC-MS SupraSolv)1:1:2 (v/v/v) in ASE (Accelerated Solvent Extraction). After that, an extract was cleaning on the column filled by silica gel (J.T. Baker) with sulphuric acid (Merck, Suprapur). The sample was reconstituted to 1 cm^3^ and measured by GC-MS. LOQ was 0.01 mg kg^−1^.

The determination of content of 309 active substances classified as pesticides in muscle tissue of fish had been carried out in accordance with the method delineated in own procedure SFFET-10. Pesticides were extracted from 2.5 g of wet tissue with MTBE (methyl t-butyl ether) (Sigma-Aldrich, Supelco) in Soxhlet/Microwave. Then the sample was separated using gel permeation chromatography (GPC) (Bio Beads SX 3, Bio-Rad, Knauer) acc. to elution profile. The solvents were evaporated to dryness. Dry residue was resolved in ethyl acetate (Merck for GC-MS SupraSolv). After cleaning with PSA (primary secondary amine, Sigma Aldrich) and centrifuging the samples, 1 cm^3^ of the supernatant was transferred to a vial and measured using GC-MS (FDP). LOQ depending on pesticide varied from 0.005 to 0.1 mg kg^−1^.

Quality Assurance

The studies were carried out by the Laboratory of the Maritime Institute, which has accreditation by the Polish Center for Accreditation. Quality Assurance of performed analyses was made using solutions with added analytical standard, analyses of CRM (Certified Reference Material) such as SPXPR-1, -3, -4, -5, -6, -10 produced by SPEXCertiPrep, 89432 TraceCERT (Glyphosate) produced by Sigma-Aldrich, IRMM-44-3 (EUROSOIL 3) and IRMM-44-4 (EUROSOIL 4) both produced by European Commission Directorate General Joint Research Centre. Quality guarantee was checked also in Proficiency Testing organised by LGC (AQUACHECK), Eurofins Scientific (SILESIALAB) and PT Pesticides in Soil (Sigma-Aldrich).

Interviews with farmers were also conducted to determine the type and amount of plant protection products used and the types of plants grown on the studied plots. In these works, a questionnaire prepared specifically for the study was used.

## Results

Farms G1 (with plot Dz 1) and G2 (with plots Dz 2 and Dz 3) covered by the study, were using in 2018 respectively 8 and 9 different types of plant protection products. On the G1 farm, the average consumption of active substances of plant protection products amounted to 0.55 kg ha^−1^ per arable land, while in the G2 farm 1.56 kg ha^−1^ per arable land. List of plant protection products used in the studied farms in the year 2018 is presented in Supplement. The farmers also declared the use of active substances propamocarb hydrochloride and fenamidone (PYTON CONSENTO 450 SC), classified as dangerous to the environment and a fungicide (AFLEX SUPER 450 SC), the active substance of which is fluopicolide. In Poland the fungicide fluopicolide has been removed from the register of plant protection products by the Minister of Agriculture and Rural Development (updated on 03.06.2019) in June 2018. Also, an organophosphorus insecticide—chlorpyrifos-ethyl belongs to compounds banned from use in fruit plants (Act on Plant Protection of December 18, 2003, Journal of Laws 2004 No. 11, item 94).

In all analysed water samples (surface and underground), soil and sediment samples, and fish tissue the concentration of organochlorine pesticides (aldrin, dieldrin, endrin, isodrine, DDT and its isomers and alpha, beta, gamma and delta isomers of hexachlorocyclohexane) were below the limit of detection of the used methodology.

However, in soil samples, surface waters from drainage ditches and watercourses as well as in groundwater samples out of 309 active substances examined, the following pesticides have been detected:

• organophosphatic insecticides: chlorpyrifos-ethyl
• fungicides: boscalid, epoxiconazole, difenoconazole, fluopicolide
• herbicides: dimetachlor, diflufenican, metazachlor, glyphosate and its metabolite AMPA
• repellents: anthraquinone.

The presence of the largest number of residues of analysed active substances was found in soil and surface water samples from drainage ditches taken from the agricultural plot Dz 1 (Table 2). The concentrations of detected pesticides in soil samples ranged from 0.05 to 0.35 mg kg^−1^. The highest concentrations were obtained in August in water from drainage ditches for: metazachlor (2.0 µg dm^−3^), glyphosate (4.7 µg dm^−3^) and AMPA (2.0 µg dm^−3^).

**Table 2:** Residues of plant protection products detected in samples taken from and around the agricultural plot No. Dz 1, and the Puck Bay. < concentration below LOQ (limit of quantification).

Month of sampling (2018)	**Plant protection product**	**Soil**	**Sediment**	**Surface water**			**Groundwater**
				**Drainage ditches**	**Bladzikowski Stream**	**The Puck Buy**	**Piezometer No. 8**
		**mg kg**^−1^		**µg dm**^−3^			
April	Chlorpyrifos-ethyl	0.093 ± 0.047	<0.015	<0.10	<0.10	<0.10	<0.10
	AMPA	0.110 ± 0.055	<0.05	<0.02	<0.02	<0.02	<0.02
June	Anthraquinone	0.18 ± 0.09	<0.06	<0.1	<0.1	<0.1	<0.1
	Boscalid	<0.09	<0.09	0.150 ± 0.038	<0.03	<0.03	<0.03
	Chlorpyrifos-ethyl	0.150 ± 0.008	<0.015	<0.10	<0.10	<0.10	<0.10
	Dimethachlor	<0.03	<0.03	0.130 ± 0.033	<0.1	<0.1	<0.1
	Epoxiconazole	<0.12	<0.12	0.170 ± 0.043	<0.03	<0.03	<0.03
	Metazachlor	<0.03	<0.03	0.12 ± 0.03	<0.03	<0.03	<0.03
	Glyphosate	0.28 ± 0.14	<0.05	<0.02	<0.02	<0.02	<0.02
	AMPA	0.35 ± 0.17	<0.05	<0.02	<0.02	<0.02	<0.02
July	Anthraquinone	0.23 ± 0.12	<0.06	<0.10	<0.10	<0.10	<0.10
	Chlorpyrifos ethyl	0.150 ± 0.008	<0.015	<0.10	<0.10	<0.10	<0.10
	Diflufenican	<0.03	<0.03	<0.03	0.130 ± 0.033	<0.03	<0.03
	Glyphosate	0.050 ± 0.025	<0.05	<0.02	<0.02	<0.02	<0.02
	AMPA	0.26 ± 0.13	<0.05	<0.02	<0.02	<0.02	<0.02
August	Boscalid	<0.09	<0.09	0.045 ± 0.023	<0.03	<0.03	<0.03
	Diflufenican	<0.03	<0.03	0.049 ± 0.025	<0.03	<0.03	<0.03
	Epoxiconazole	<0.12	<0.12	0.045 ± 0.023	<0.03	<0.03	<0.03
	Metazachlor	<0.03	<0.03	2.0 ± 1.0	<0.03	<0.03	<0.03
	Glyphosate	<0.05	<0.05	4.7 ± 2.4	<0.02	<0.02	<0.02
	AMPA	<0.05	<0.05	2.0 ± 1.0	<0.02	<0.02	<0.02

Concentrations of residues of plant protection products found in samples taken from the area of plot Dz 2 and in its vicinity are presented in Table 3. In Table 4 the results obtained in samples taken from and around the area of plot Dz 3 are showed. Both plots belong to farm G2. As a result of the conducted research, the presence of fluopicolide in soils (0.15 mg kg^−1^) and waters of drainage ditches (0.37 µg dm^−3^) was found in July on plot Dz 3. In the waters of drainage ditches fluopicolide was found also in August on plot Dz 2 (0.02 µg dm^−3^). On the G2 farm, plant protection products containing fluopicolide and glyphosate were used also in 2017. In July, the presence of diflufenican in the surface waters of Bladzikowski Stream was also noted. Part of the drainage ditches surrounding agricultural farms falls into this stream.

**Table 3:** Residues of plant protection products detected in samples taken from and around the agricultural plot No. Dz 2, and the Puck Bay. < concentration below LOQ (limit of quantification).

**Month of sampling** (2018)	**Plant protection product**	**Soil**	**Sediment**	**Surface water**			**Groundwater**
				**Drainage ditches**	**Bladzikowski Stream**	**The Puck Buy**	**Piezometer No. 31**
		**mg kg**^−1^		**µg dm**^−3^			
March	AMPA	0.150 ± 0.075	<0.05	<0.02	<0.02	<0.02	<0.02
June	AMPA	0.20 ± 0.10	<0.05	<0.02	<0.02	<0.02	<0.02
July	Glyphosate	0.050 ± 0.025	<0.05	<0.02	<0.02	<0.02	<0.02
	AMPA	0.170 ± 0.085	<0.05	<0.02	<0.02	<0.02	<0.02
	Diflufenican	<0.03	<0.03	<0.03	0.130 ± 0.033	<0.03	<0.03
August	Fluopicolide	<0.03	<0.03	0.020 ± 0.010	<0.02	<0.02	<0.02
	Metazachlor	<0.03	<0.03	0.037 ± 0.019	<0.03	<0.03	<0.03
	Glyphosate	<0.05	<0.05	0.170 ± 0.085	<0.02	<0.02	<0.02
	AMPA	<0.05	<0.05	0.20 ± 0.010	<0.02	<0.02	0.110 ± 0.055

**Table 4:** Residues of plant protection products detected in samples taken from and around the agricultural plot No. Dz 3, and the Puck Bay. < concentration below LOQ (limit of quantification).

**Month of sampling** (2018)	**Plant protection product**	**Soil**	**Sediment**	**Surface water**			**Groundwater**
				**Drainage ditches**	**Bladzikowski Stream**	**The Puck Buy**	**Piezometer No. 31**^1^ & **56**^2^
		**mg kg**^−1^		**µg dm**^−3^			
June	AMPA	<0.05	<0.05	<0.02	<0.02	<0.02	<0.02
July	Difenoconazole	0.20 ± 0.10	<0.045	<0.10	<0.10	<0.10	<0.10
	Fluopicolide	0.150 ± 0.074	<0.03	0.370 ± 0.093	<0.02	<0.02	<0.02
	AMPA	0.069 ± 0.035	<0.05	<0.02	<0.02	<0.02	<0.02
August	Fluopicolide	<0.03	<0.03	0.020 ± 0.010	<0.02	<0.02	<0.02
	Glyphosate	<0.05	<0.05	0.170 ± 0.085	<0.02	<0.02	0.021 ± 0.105^2^
	AMPA	<0.05	<0.05	0.20 ± 0.010	<0.02	<0.02	0.110 ± 0.055^1^

Glyphosate and AMPA in surface water samples taken from drainage ditches surrounding studied plots were found in period after intensive use of pesticides in concentrations between 0.17–4.7 µg dm^−3^ and 0.2–2.0 µg dm^−3^ respectively.

Both glyphosate and AMPA in groundwater were found only in one out of four samples in concentrations 0.021 µg dm^−3^ and 0.11 µg dm^−3^ respectively.

Glyphosate and AMPA were found in soil samples taken from all studied plots mainly in the period of intensive application of PPP. Detected concentrations of glyphosate and AMPA varied between 0.069–0.35 mg kg^−1^ and 0.05–0.28 mg kg^−1^ respectively.

In the surface water samples and sediment samples taken from the Bay of Puck, none of the 309 substances from the pesticides group were found.

## Discussion

The concentration of organochlorine pesticides (aldrin, dieldrin, endrin, isodrine, DDT and its isomers and alpha, beta, gamma and delta isomers of hexachlorocyclohexane) were below the limit of detection of the used methodology as well as below the limit values in surface waters established for particular compounds (Annex 9—Environmental quality standards for priority substances and for other pollutants, Journal of Laws of 2016, item 1878). It is confirmed by the results of pesticides obtained in monitoring the chemical status of groundwater in Poland. The concentration of individual compounds from organochlorine pesticides in the majority of measurement points did not exceed the limit of detection (Cabalska et al., 2015). Exceedances of priority substances (occurring in plant protection products or used for their production) were not detected in the framework of the State Environmental Monitoring covering, inter alia, surface and underground water, either Ministry of Agriculture and Rural Development (2018).

The presence of the largest number of residues of pesticides (chlorpyrifos-ethyl, anthraquinone, boscalid, dimetachlor, epoxiconazole, diflufenican, metazachlor, glyphosate, AMPA) was found in soil and surface water samples from drainage ditches taken from the agricultural plot Dz 1. The concentrations of metazachlor, glyphosate and AMPA in water from drainage ditches were obtained in August. It may be the result of poor mobility of these active substances and weather conditions, namely drought in July and more intense precipitation in August, which probably washed away the tested substances from soils. The risk of off-site airborne transport of glyphosate and AMPA with dust is very high, because glyphosate and AMPA hardly decay under dry conditions of the soil (Bento et al., 2017).

Das et al. (2019) stated that wind transport and wind direction played a significant role in distribution of chlorpyrifos. They found concentrations of chlorpyrifos in air up to 500 m from the field at levels considered concerning for human health. The degradation rate of several pesticides (among others diflufenican) has been found to be related to pH of soils (pH lower than 6.5 showed slower co-metabolic degradation) (Houot et al., 2000; Bending, Lincoln & Edmonson, 2006).

Our study indicated that glyphosate and AMPA contents were found in most of analysed matrices: soil, surface water from drainage ditches and streams, and groundwater.

Glyphosate is currently the most commonly used herbicide in agricultural lands worldwide (Battaglin et al., 2014; Sidoli, Baran & Angulo-Jaramillo, 2016; Bento et al., 2017; Erban et al., 2018; Gunarathna et al., 2018; Lupi et al., 2019). The license for its use was extended by the European Commission until 15th December 2027. However, the European Parliament is seeking to ban the substance in households immediately and to gradually reduce its application in agriculture, until a total ban after 2022 (Weber & Burtscher-Schaden, 2019). Glyphosate and AMPA in surface water samples taken from drainage ditches surrounding studied plots were found in period after intensive use of pesticides. The concentrations of glyphosate and AMPA obtained in the study varied between 0.17–4.7 µg dm^−3^ and 0.2–2.0 µg dm^−3^ respectively. Battaglin et al. (2014) in ditches and drains from USA area were detected glyphosate in median concentration 0.20 µg dm^−3^ (max 427 µg dm^−3^) and AMPA in median concentration 0.43 µg dm^−3^ (max 397 µg dm^−3^). Sidoli, Baran & Angulo-Jaramillo (2016) concluded that analysis in drainage-water samples underscored the possible leaching of glyphosate and AMPA through the soil and thus the risk of groundwater contamination.

In the present study both glyphosate and AMPA in groundwater were found only in one out of four samples in concentrations 0.021 µg dm^−3^ and 0.11 µg ⋅dm^−3^ respectively. Gunarathna et al. (2018) were detected glyphosate and AMPA in groundwater of Sri Lanka in concentrations 1–4 µg dm^−3^ and 2–11 µg dm^−3^ respectively. Battaglin et al. (2014) were detected content of glyphosate in groundwater from USA area less than 0.2 µg dm^−3^(median) and 2.03 µg dm^−3^(max), and AMPA: less than 0.2 µg dm^−3^(median) and 4.88 µg dm^−3^(max).

Glyphosate and AMPA were found in soil samples taken from all studied plots mainly in the period of intensive application of pesticides. Detected concentrations of glyphosate and AMPA varied between 0.069–0.35 mg kg^−1^ and 0.05–0.28 mg kg^−1^ respectively. Battaglin et al. (2014) also reported content of glyphosate and AMPA in soils from USA area on similar levels: median concentration of glyphosate 0.010 mg kg^−1^ (max 0.48 mg kg^−1^) and median concentration of AMPA 0.018 mg kg^−1^ (max 0.34 mg kg^−1^). Gunarathna et al. (2018) were detected glyphosate and AMPA in soils (Sri Lanka) in concentrations 0.27–0.69 mg kg^−1^ and 0.002–0.008 mg kg^−1^ respectively. Higher concentrations were detected in soils from Belgium (Huldenberg): 5.5–16 mg kg^−1^ (glyphosate) and 0.07–0.7 mg kg^−1^ (AMPA) (Bento et al., 2017).

In the surface water samples and sediment samples taken from the Bay of Puck, none of the 309 substances from the pesticides group were found. However, in studies conducted in water samples from ten German Baltic estuaries the presence of glyphosate and AMPA were confirmed. Concentrations ranges observed were 0.028 to 1.69 µg dm^−3^ and 0.045 to 4.156 µg dm^−3^ for glyphosate and AMPA respectively (Skeff, Neuman & Schulz-Bull, 2015).

In the conducted studies, no active substances were found in the muscle tissue of the analysed cod and flounder. However, pesticide residues are detected in various fish species. Research carried out by Oliveira et al. (2015) showed the presence of organophosphorus insecticides in fish caught in the São Francisco River in Brazil. In this study chlorpyrifos, dichlorvos, diazinon, disulfonone, etrinphos, etion, phosmet, phosalone and pyrazophos were detected. These compounds were found both in internal organs and in fish muscles. Chlorpyrifos (36.1%) and dichlorvos (33.3%) were present in the largest amounts. Dichlorvos is widely used in fish farming to control external parasites. In fish exposed to long-term effects of pesticides, a number of changes in the blood and interior organs are observed. Among others, a decrease in the activity of lysozyme and globulins in plasma of rainbow trout, at stated concentration of diazinon 0.1–0.2 mg dm^−3^ was observed (Banaee et al., 2011; Ahmadi et al., 2014). In contrast, chlorpyrifos already in a concentration of 0.015 mg dm^−3^ was the cause of increased activity of lysozyme and immunoglobulin M in the kidneys and decrease of M immunoglobulin in the carp spleen. At a concentration of 0.04 mg dm^−3^, chlorpyrifos also increased the number of white blood cells (Witczak & Pohoryło, 2016).

## Conclusions

In the analysed water samples (surface, underground and marine), soil and sediment samples, and fish muscle, organochlorine insecticides (aldrin, dieldrin, endrin, isodrine, DDT and its isomers and alpha, beta, gamma and delta isomers of hexachlorocyclohexane), were not detected. Organochlorine pesticides are designated for research in the Polish State Environmental Monitoring and for which limit values in environmental matrices are defined. Presence of glyphosate and its metabolite AMPA, anthraquinone, boscalid, chlorpyrifos-ethyl, dimethachlor, diflufenican, difenoconazole, epoxiconazole, fluopicolide and metazachlor was found in soil samples and surface water samples collected from drainage ditches surrounding the studied agricultural plots. In addition to glyphosate, fluopicolide and chlorpyrifos, substances detected in the tested samples are not active substances of pesticides, the use of which has been revealed by the farmers in the questionnaire. In one case the use of chlorpyrifos-ethyl was declared by the farmer in 2017. It is a substance showing poor mobility in soil and its remains were probably detected in studies carried out in 2018. The largest number of residues of pesticides was found in soil and surface water samples from drainage ditches taken from the agricultural plot Dz 1. Here, in August the highest concentrations of metazachlor, glyphosate and its metabolite AMPA were found, also. The reason may also be the terrain, which in the examined Puck commune area is characterized by slopes of up to approximately 9%, which favours leaching of the applied plant protection products from soils as a result of surface runoff (washing and running) and transferring them to other fields. In April, June and July, the presence of active substances was found in soil samples. In surface water collected from drainage ditches surrounding the plot, active substances appeared in June following atmospheric precipitation. There are no legislative limits set for analysed active substances in environmental matrices such as water and soil. Results confirming the presence of glyphosate and AMPA also in groundwater appear to warrant the complete ban on its use, because of risks it poses to health and life of people.

As part of the conducted tests, in the samples of surface water, sediment and fish taken from the Puck Bay there was no presence of glyphosate and AMPA, or any of the 309 substances tested in the group of pesticides, either. The amount of active substances of plant protection products that flows from the surface of arable land with watercourses and ditches, followed by canals and rivers to the Puck Bay is insignificant, and in its waters is diluted to undetectable levels. However, attention should be paid to the farmer’s exercise of good plant protection practices in order to further minimize the negative impact of pesticides on the environment of the Bay of Puck, especially due to the fact that the quality of food (e.g., fish) is closely related to the place of its acquisition and the state of the environment. Even low concentrations of pesticides in food can cause negative health effects, especially when exposure is extended over time. Thus, examination of active substances of the plant protection products in the environment seems to be necessary.

##  Supplemental Information

10.7717/peerj.8789/supp-1 Supplemental Information 1 Raw data: pesticides in soil

10.7717/peerj.8789/supp-2 Supplemental Information 2 Raw data: pesticides in rivers

10.7717/peerj.8789/supp-3 Supplemental Information 3 Raw data: pesticides in drainage ditches

10.7717/peerj.8789/supp-4 Supplemental Information 4 Raw data: pesticides in groundwater

10.7717/peerj.8789/supp-5 Supplemental Information 5 Residues of plant protection products detected in samples taken from and around the agricultural parcel No. Dz 3, and the Puck Bay Concentration below LOQ (limit of quantification)

## Additional Information and Declarations

### Field Study Permissions

The following information was supplied relating to field study approvals (i.e., approving body and any reference numbers): Sampling were carried out with the consent of the Puck commune and farmers who took part in the project.

### Data Availability

The following information was supplied regarding data availability: Anonymized raw data are available in the Supplemental Files.
