# Supplementary material for: The impact of pesticides used at the agricultural land of the Puck commune on the environment of the Puck Bay
Source: PeerJ. 2020 Mar 19;8:e8789. doi: 10.7717/peerj.8789 (PMC7085897; doi:10.7717/peerj.8789)
Supplement: Supplemental Information 5 — Concentration below LOQ (limit of quantification) [file peerj-08-8789-s005.docx]

Supplement: List of plant protection products used in the studied farms in the year 2018

| **Use of a plant protection product** | **Name of the plant protection product according to the MA&RD register ^1)^** | **Active substance No. 1** | | **Active substance No. 2** | | **Active substance No. 3** | |
| --- | --- | --- | --- | --- | --- | --- | --- |
|  |  | **name** | **content** | **name** | **content** | **name** | **content** |
| **Farm No. G1** | | | | | | | |
| Herbicide for monocotyledonous and dicotyledonous weeds in winter wheat, winter triticale and rye | HUZAR ACTIV 387 OD | iodosulfuron methylsodium | 10 g⋅dm^-3^ | 2,4-D | 377 g⋅dm^-3^ |  |  |
| Fungicide in the form of a concentrate for making a water emulsion, with a deep-seated action, for preventive and interventive use in the protection of winter wheat | FAXER 450 EC | prochloraz | 450 g⋅dm^-3^ |  |  |  |  |
| Herbicide used in the cultivation of potato, winter rapeseed, spring rapeseed, peas, beans, carrots, cucumbers, celery root | COMMAND 480 EC | clomazone | 480 g⋅dm^-3^ |  |  |  |  |
| Herbicide used for the control of dicotyledonous weeds and certain monocot weeds in potato cultivation | ELAFI 70 WG | metribuzin | 700 g⋅kg^-1^ |  |  |  |  |
| Fungicide used in the protection of potato, tomato, onion and cucumber | EKONOM MC 72.5 WP | mancozeb | 680 g⋅kg^-1^ | cymoxanil | 45 g⋅kg^-1^ |  |  |
| Fungicide, concentrate for diluting with water, deep-seated and systemic action for preventive and interventive use in potato protection against potato blight | PROXANIL | propamocarb hydrochloride | 400 g⋅dm^-3^ | cymoxanil | 50 g⋅dm^-3^ |  |  |
| Insecticide used in early spring treatments against stem pests in winter rapeseed | INAZUMA 130 WG | acetamiprid | 100 g⋅kg^-1^ | lambda-cyhalothrin | 30 g⋅kg^-1^ |  |  |
| Herbicide intended for the control of couch grass and other monocot and dicotyledonous weeds (annual and perennial) before harvesting winter wheat, winter barley to control weeds and accelerate harvesting, before harvesting winter rapeseed to accelerate harvesting, weed control and management of fallow land before sowing crops | GALLUP 360 SL | **glyphosate ^4)^** | 360 g⋅dm^-3^ |  |  |  |  |
| **Farm No. G2** | | | | | | | |
| Herbicide used in the protection of winter wheat, winter triticale, rye | LANCET PLUS 125 WG | florasulam | 25 g⋅kg^-1^ | piroxsulam | 50 g⋅kg^-1^ | aminopyralid | 50 g⋅kg^-1^ |
| Fungicide used in the protection of wheat, triticale, barley and rye and sugar beet | SOPRANO 125 SC | **epoxiconazole ^4)^** | 125 g⋅dm^-3^ |  |  |  |  |
| Herbicide applied to dicotyledonous weeds and some monocotyledonous in winter rapeseed | COLZOR TRIO 405 EC | **dimethachlor ^4)^** | 187.5 g⋅dm^-3^ | napropamide | 187.5 g⋅dm^-3^ | clomazone | 30 g⋅dm^-3^ |
| Herbicide used in the cultivation of potato, winter rapeseed, spring rapeseed, peas, beans, carrots, cucumbers, celery root | COMMAND 480 EC | clomazone | 480 g⋅dm^-3^ |  |  |  |  |
| Herbicide used in potatoes, corn, carrots and other vegetables, soy, peas, lupine, sunflower, herbs | **AFALON DYSPERSYJNY 450 SC ^2)^** | linuron | 450 g⋅dm^-3^ |  |  |  |  |
| Fungicide for protection against fungal diseases of potato, vine, tobacco, cultivated in the soil: tomato, onion, broccoli, cauliflower, lettuce, ornamental plants and grown under covers: cucumber and ornamental plants | RIDOMIL GOLD MZ PEPITE 67.8 WG | metalaxyl - M | 38.8 g⋅kg^-1^ | mancozeb | 640 g⋅kg^-1^ |  |  |
| Herbicide used to control one-year dicotyledonous weeds in potato, faba bean, fodder pea, pea and carrot | **AFLEX SUPER 450 SC ^2)^** | linuron | 450 g⋅dm^-3^ | **fluopicolide ^4)^** | 62.5 g⋅dm‑^3^ |  |  |
| Fungicide used in the protection of potato and tomato in cultivation under covers | **PYTON CONSENTO 450 SC ^3)^** | **propamocarb hydrochloride** | **375 g⋅dm^-3^** | **fenamidone** | **75 g⋅dm^-3^** |  |  |
| Herbicide for post-emergence control of annual and perennial dicotyledonous weeds in spring barley, winter barley, spring wheat, winter wheat, winter triticale, spring triticale, rye, oats, cereal mixes | **LINTUR 70 WG ^2)^** | dicamba | 659 g⋅kg^-1^ | triasulfuron | 41 g⋅kg^-1^ |  |  |

Notes.

(1) The register of plant protection products authorized by Minister of Agriculture and Rural Developmentupdate 2019.06.03. (2) The substance removed from the register of plant protection products in June 2018. (3) A substance dangerous for the environment according to the classification of a plant protection product in terms of its environmental
hazards. (4) Active substances detected in the tested samples.
